# Clinical Consequences of Inadvertent Intramuscular Administration of Subcutaneous Risperidone Formulation

**DOI:** 10.1155/crps/7485684

**Published:** 2026-09-09

**Authors:** Emma Ashley, Arnela Aalomerovic, Kyle Blackmer, Rif S. El-Mallakh

**Affiliations:** ^1^ Mood Disorders Research Program, Depression Center, Department of Psychiatry and Behavioral Sciences, University of Louisville School of Medicine, Louisville, Kentucky, USA, louisville.edu

## Abstract

Long‐acting injectable (LAI) antipsychotics have been predominately administered intramuscularly (IM). Recently, subcutaneous (SC) formulations have been developed. Information regarding what occurs when the drug is not administered properly is lacking. A 23‐year‐old woman with bipolar disorder, currently manic with psychosis, was responding well to a combination of lithium and risperidone. She accepted a LAI of SC risperidone, but the drug was administered IM by error. The improvement she had already displayed was partially lost, and oral risperidone had to be reinitiated. She complained of mild pain at the injection site, but no other adverse consequences were noted. When a SC formulation is administered IM, it results in mild muscle pain, and unpredictable release of risperidone, but no other notable clinical problems.

## 1. Introduction

Long‐acting injectable (LAI) antipsychotic medications offer many clinical advantages to patients with schizophrenia or bipolar disorder [1]. Intramuscular and subcutaneous (SC) formulations are available. Surveys suggest that a majority of both clinicians (65%) and patients (57%) preferred SC injections [2]. Pathological examination of the injection site in animals receiving SC or intramuscularly (IM) LAI aripiprazole revealed that both routes are associated with a local inflammatory reaction, which was more pronounced with SC [3]. Nonetheless, the SC route has a lower risk of injury to muscle, nerves, blood vessels, and deeper structures and puts forward the possibility of self‐administration in some cases. There are no reports of outcomes of administration of formulations intended for one route when they are used in an alternate tissue. Medication errors occur in approximately 6.5% of hospitalized patients [4], and investigating them can serve to educate clinicians regarding outcomes of these errors (e.g., [5]). Herein, we report a case of inadvertent intramuscular administration of a SC formulation of risperidone.

## 2. Case Report

A 23‐year‐old female college student with a history of bipolar disorder type I was admitted to a psychiatric unit for a manic episode with hallucinations, thought disturbance, and a reduced need for sleep. She was treated with oral lithium carbonate (ultimately 1200 mg/day, level 1.1 mEq/L), lamotrigine (ultimately 100 mg/day), and risperidone (ultimately 4 mg/day). She was showing significant improvement by the sixth day of hospitalization and agreed to receive a SC long‐acting risperidone injection with a plan to begin to taper and stop the oral dose the following day. On the sixth day of hospitalization, Uzedy 100 mg was accidentally administered in the left deltoid, and oral risperidone was reduced to 2 mg on the seventh day. The discovery of this error occurred on the seventh day of hospitalization. She reported mild discomfort at the injection site beginning the same day and persisting till discharge 10 days later. Her symptoms got worse after tapering the oral risperidone dose to 2 mg, with worsening of thought disorganization, necessitating reincrease to 4 mg, which was continued until discharge. There were no significant changes in any of her vital signs (around 130/75, pulse 90 s, and O_2_ saturation of 100%) or laboratory investigations (complete metabolic profile and complete blood count), which all remained normal, as with the electrocardiogram (ECG) with a QT_c_ of 447 msec 1 week after the shot.

She was discharged off oral risperidone 1 week after the IM shot with plans to continue with the SC risperidone 28 days after the initial injection. There were no additional complaints or problems.

The University of Louisville’s Human Subjects Protection Program does not review individual case reports. However, prior to discharge, the patient agreed to have her case written up for publication and provided written informed consent.

## 3. Discussion

The two most notable occurrences after inadvertent administration of the SC formulation into the deltoid muscle were muscle pain and worsening of her psychotic symptoms. We did not measure risperidone levels, so it is possible that reduction of the oral dose was responsible for the worsening of the symptoms, although therapeutic plasma levels are reached within 24 h [6] of SC administration. Alternatively, we may have been observing variations in the symptoms as the patient was slowly improving.

SC risperidone formulations use a copolymer that becomes a solid after SC formulation. The Uzedy formulation is proprietary but utilizes repeating chains of hydrophilic polyethylene glycol surrounded by hydrophobic polyester chains that precipitate into a solid when exposed to water, trapping the risperidone within [1, 7]. The immediate release of risperidone, which results in nearly immediate therapeutic levels, occurs when some of the drug, which is mixed within the polymers in a solvent, is carried away into the body, while the entrapped risperidone is released slowly as the polymers breakdown into nontoxic chemicals that the body can manage easily. These chemicals are related to those used for dissolvable sutures [8], which are known to cause inflammation [9]. Our patient did not have any induration, tenderness, or redness at the injection site. Nonetheless, it is possible that the muscle pain described by the patient was related to, at least in part, inflammation caused by the administered drug.

It is important to note that when medications intended for SC administration, for example, insulin, are administered IM, the absorption is unpredictable although generally faster [10]. Pain is clearly greater with IM versus SC injection of insulin [10]. Similarly, dissolvable suture will dissolve faster in acidic environments (like the muscle, where the pH decreases to 7.1 with activity [11]) versus SC tissues (where the pH is generally around 7.4 [12]) [13]. Our case suggests that absorption from the muscle may be less predictable, but there is no evidence that it was faster.

Finally, it is important to note that nurses generally expect LAI antipsychotics to be administered IM [14]. This was probably the root cause of our medication error. Given the larger variety of LAI injectables now available (IM versus SC, longer and more varied intervals, and a variable period of time for the next injection if a shot is missed), training can reduce the risk of medication errors related to LAI [15].

In this case, intramuscular administration of a SC formulation of risperidone was associated with a poorer than anticipated response to risperidone and some mild pain in the muscle. There were no other observed adverse untoward consequences. In similar situations, clinicians should note any local adverse effects, carefully monitor psychiatric symptoms, and taper oral antipsychotic medications accordingly.

## Funding

No funding was received for this study.

## Conflicts of Interest

Rif S. El‐Mallakh is a speaker for Axsome, Johnson and Johnson, Lundbeck, Otsuka, and Vanda. The other authors declare no conflicts of interest.

## Data Availability

The data that support the findings of this study are available from the corresponding author upon reasonable request.
